# Associations Between Early Neurosurgical Workflow and Survival in Primary Central Nervous System Lymphoma: A Single-Center Retrospective Study

**DOI:** 10.3390/curroncol33030139

**Published:** 2026-02-27

**Authors:** Emre Ozkara, Eray Horoz, Zuhtu Ozbek, Deniz Arik, Funda Canaz, Suzan Saylisoy, Hava Uskudar Teke, Murat Vural

**Affiliations:** 1 Department of Neurosurgery, Eskisehir Osmangazi University Faculty of Medicine, Eskisehir 26480, Türkiye; eray.horoz@ogu.edu.tr (E.H.); zozbek@ogu.edu.tr (Z.O.); mvural@ogu.edu.tr (M.V.); 2 Department of Pathology, Eskisehir Osmangazi University Faculty of Medicine, Eskisehir 26480, Türkiye; darik@ogu.edu.tr (D.A.); fcanaz@ogu.edu.tr (F.C.); 3 Department of Radiology, Eskisehir Osmangazi University Faculty of Medicine, Eskisehir 26480, Türkiye; ssaylisoy@ogu.edu.tr; 4 Department of Internal Medicine, Division of Hematology, Eskisehir Osmangazi University Faculty of Medicine, Eskisehir 26480, Türkiye; huteke@ogu.edu.tr

**Keywords:** primary central nervous system lymphoma, neurosurgical workflow, corticosteroids, diagnostic delay, biopsy timing, survival analysis

## Abstract

Primary central nervous system lymphoma (PCNSL) is an aggressive malignancy for which early management decisions frequently occur within neurosurgical workflows prior to oncologic treatment. In this retrospective single-center study, we aimed to explore whether early neurosurgical workflow characteristics are associated with survival outcomes in patients with PCNSL. Consecutive adult patients diagnosed with PCNSL between 2012 and 2022 were included, and the variables of interest comprised pre-biopsy corticosteroid exposure, the interval between diagnostic magnetic resonance imaging (MRI) and stereotactic biopsy, and the time from biopsy to initiation of high-dose methotrexate–based induction therapy. All patients were treated under a standardized hematology protocol to limit systemic treatment heterogeneity. Overall survival (OS) and progression-free survival (PFS) were calculated from the date of diagnostic biopsy, and survival analyses were performed using Kaplan–Meier methods and log-rank testing. Twenty-nine patients met the inclusion criteria. Median OS and PFS were not reached in steroid-naïve patients, whereas pre-biopsy corticosteroid exposure was associated with consistently shorter survival trajectories, with a clear separation of the survival curves, despite conventional statistical significance not being reached. Similarly, median OS and PFS were not reached in patients undergoing biopsy within 7 days of MRI, and an MRI-to-biopsy interval exceeding 7 days demonstrated an unfavorable survival trajectory compared with earlier biopsy; biopsy-to-induction timing did not show a measurable association with early survival outcomes. Established prognostic stratification using Memorial Sloan–Kettering Cancer Center classes showed expected survival discrimination within the cohort, supporting internal validity. Given the limited sample size and retrospective design, all findings should be interpreted as exploratory associations rather than evidence of causality. These results suggest that early neurosurgical workflow characteristics, particularly empiric pre-biopsy corticosteroids avoidance and diagnostic delay minimization, may be associated with early survival trajectories in PCNSL and warrant further evaluation in larger prospective studies.

## Simple Summary

Primary central nervous system lymphoma (PCNSL) is a rare but aggressive brain tumor for which critical clinical decisions are often made before patients enter formal oncologic care. In routine practice, neurosurgeons frequently control the early diagnostic phase through corticosteroid administration and biopsy timing. In this study, we explore whether these early neurosurgical workflow decisions are associated with patient survival, observing that pre-biopsy corticosteroid exposure and delays between MRI and biopsy are associated with less favorable survival trajectories, with a clear separation of survival patterns despite the absence of conventional statistical significance. Although limited by sample size, these findings suggest that the earliest phase of care may represent a modifiable window with potential prognostic relevance. Increased interdisciplinary awareness of this early diagnostic period may contribute to improved PCNSL management strategies.

## 1. Introduction

Primary central nervous system lymphoma (PCNSL) is an aggressive, extranodal, non-Hodgkin lymphoma confined to the brain, leptomeninges, eyes, or spinal cord [1]. Although high-dose methotrexate (HD-MTX)-based chemotherapy remains the standard treatment, survival outcomes depend heavily on timely diagnosis and early initiation of therapy [1,2,3]. In real-world settings, however, patients rarely present with PCNSL initially to hematology services; they typically first encounter neurology or neurosurgery clinics, presenting with nonspecific symptoms such as cognitive impairment, headache, confusion, seizures, or focal deficits [4,5].

Because PCNSL is uncommon in neurosurgical practice, early management frequently mirrors workflows used for gliomas or inflammatory lesions. This mismatch is clinically important because, unlike low-grade gliomas, PCNSL exhibits rapid proliferation and radiographic evolution, making short diagnostic delays potentially harmful [3,6,7].

Pre-biopsy corticosteroid exposure has been shown to induce radiographic regression, obscure histopathology, and exert cytotoxic pressures that can reshape clonal architecture [4,8,9]. Similarly, delays between MRI and biopsy, often dictated by neurosurgical workflow, bed availability, or consultation patterns, create biologically active intervals during which lymphoma growth continues unchecked [10,11].

Despite these factors, major prognostic models (MSKCC, IELSG) primarily emphasize host- and treatment-related variables and largely overlook the neurosurgical entrance phase [12,13,14], so the question of whether early, modifiable neurosurgical workflow decisions have measurable survival relevance remains insufficiently studied.

The central focus of this study is to investigate whether the neurosurgical entry point, prior to the commencement of oncological therapy, is a biologically active and modifiable factor that influences early survival outcomes in patients with primary central nervous system lymphoma (PCNSL). To minimize the impact of systemic therapy, all patients were treated under a standardized hematology protocol. In this study, we therefore aim to evaluate the relationship between neurosurgical workflow factors, such as pre-biopsy steroid exposure and diagnostic timing, and early survival outcomes in a consecutive, real-world cohort of PCNSL patients.

## 2. Materials and Methods

### 2.1. Study Design and Patient Selection

We conducted a single-center retrospective cohort study including all adult patients diagnosed with primary central nervous system lymphoma (PCNSL) at Eskişehir Osmangazi University between January 2012 and December 2022. PCNSL diagnosis was confirmed histopathologically in accordance with the 2025 update of the World Health Organization (WHO) Classification of Tumors of the Central Nervous System (5th edition) [15].

The inclusion criteria were as follows:

1. Age ≥ 18 years;
2. Histopathologically confirmed PCNSL (diffuse large B-cell lymphoma);
3. Availability of pre-biopsy brain magnetic resonance imaging (MRI);
4. Availability of recorded dates for diagnostic MRI, neurosurgical evaluation, stereotactic biopsy, and induction chemotherapy initiation;
5. Availability of clinical follow-up data for overall survival (OS) and progression-free survival (PFS).

The exclusion criteria were as follows:

1. Secondary central nervous system involvement from systemic lymphoma;
2. Multifocal disease with insufficient anatomical characterization on MRI;
3. Absence of histological confirmation due to deferred or non-diagnostic biopsy;
4. Missing survival data;
5. Receipt of definitive treatment at external institutions prior to referral.

The following section will address the clinical variables and workflow definitions.

To assess the influence of the neurosurgical workflow on outcomes, three key interval metrics were predefined based on their clinical relevance and the prior PCNSL literature.

The MRI-to-biopsy interval was defined as the number of days between the initial diagnostic MRI and stereotactic biopsy. Patients were categorized into three groups (0–7 days, 8–14 days, and ≥15 days), with these categories predefined to reflect real-world neurosurgical workflow thresholds rather than biologically validated cut-offs, corresponding to the expedited versus elective diagnostic pathways commonly encountered in clinical practice.

Pre-biopsy corticosteroid exposure was analyzed as a binary variable (any exposure vs. none). Due to the retrospective nature of this study and heterogeneity in medical documentation, the cumulative dose and duration of corticosteroid therapy could not be reliably assessed.

The biopsy-to-induction interval was defined as the time between diagnostic biopsy and initiation of high-dose methotrexate (HD-MTX)-based induction therapy and was analyzed both continuously and categorically (≤7 days vs. >7 days), reflecting clinically relevant timelines for treatment initiation.

All biopsies were performed using stereotactic or frameless techniques by board-certified neurosurgeons. Induction chemotherapy consisted of HD-MTX-based regimens administered under the supervision of hematology and oncology specialists. Variations in methotrexate dosing, number of cycles, and consolidative therapies (e.g., cytarabine or whole-brain radiotherapy) reflected real-world clinical practice.

### 2.2. Outcome Measures

Overall survival (OS) was defined as the time from diagnostic biopsy to death from any cause or last follow-up. Progression-free survival (PFS) was defined as the time from biopsy to radiological or clinical progression, relapse, or death. Patients without documented events were censored at their last known follow-up.

### 2.3. Statistical Analysis

Descriptive statistics are reported as frequencies (%) for categorical variables and as medians with ranges for time-related variables, as appropriate. Group comparisons were performed using the Mann–Whitney U test for continuous variables and the Chi-square or Fisher’s exact test for categorical variables. Survival distributions were estimated using the Kaplan–Meier method and compared using the log-rank test, and median survival times were derived directly from the Kaplan–Meier estimates. Given the limited sample size and number of survival events, multivariable Cox proportional hazards modeling was deliberately avoided to prevent unstable estimates and violations of event-per-variable assumptions. Accordingly, all analyses were considered exploratory and hypothesis-generating. A two-sided *p*-value < 0.05 was considered statistically significant, and the statistical analyses were conducted using SPSS version 27 (IBM Corp., Armonk, NY, USA).

## 3. Results

A total of 29 patients diagnosed with primary central nervous system lymphoma (PCNSL) were included in the analysis, whose baseline demographic, clinical, and workflow characteristics are summarized in Table 1. The cohort predominantly consisted of older adults, with 58.6% of patients aged ≥60 years. The Memorial Sloan–Kettering Cancer Center (MSKCC) prognostic classes were distributed as follows: Class 1 in 13.8%, Class 2 in 62.1%, and Class 3 in 24.1% of patients. Pre-biopsy corticosteroid exposure was observed in 48.3% of the cohort, and the MRI-to-biopsy interval was ≤7 days in 75.9% of patients.

**Figure 1:**
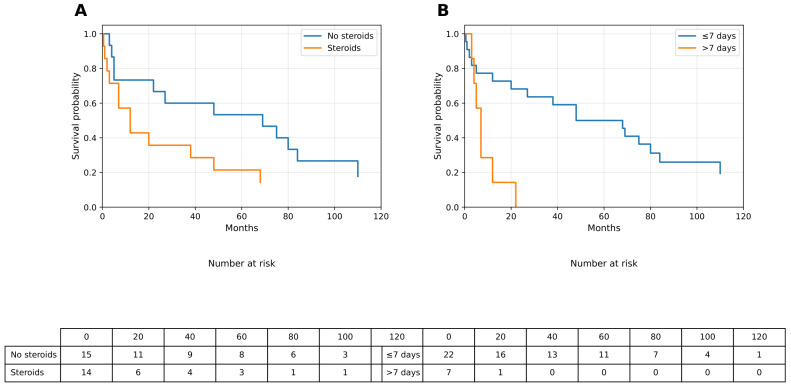
Overall survival according to neurosurgical workflow variables. (**A**) Kaplan–Meier curve of overall survival stratified by pre-biopsy corticosteroid use. (**B**) Kaplan–Meier curve of overall survival stratified by MRI-to-biopsy interval (≤7 days vs. >7 days). Median survival values were derived from Kaplan–Meier estimates. OS, overall survival; MRI, magnetic resonance imaging.

**Overall Survival According to Pre-biopsy Steroid Exposure:** Patients who received corticosteroids prior to biopsy demonstrated an unfavorable overall survival (OS) pattern compared with steroid-naïve patients; median OS was not reached in the no-steroid group, whereas the median OS was 12 months in the steroid-exposed group. Although the log-rank comparison did not reach conventional statistical significance (*p* = 0.127), Kaplan–Meier curves showed a consistent separation between the two groups throughout follow-up (Figure 1A). Detailed OS estimates are presented in Table 2.

**Overall Survival According to MRI-to-Biopsy Timing:** When stratified by diagnostic timing, patients who underwent biopsy within ≤7 days of MRI exhibited more favorable OS trajectories compared with those who experienced delays exceeding 7 days; median OS was not reached in the ≤7-day group, whereas patients with an MRI-to-biopsy interval >7 days had a markedly shorter survival, with median OS ranging between 5 and 7 months. The log-rank test demonstrated a directional trend toward reduced OS with increasing diagnostic delay (*p* = 0.074), and corresponding Kaplan–Meier curves are shown in Figure 1B, with numerical values summarized in Table 2.

**Figure 2:**
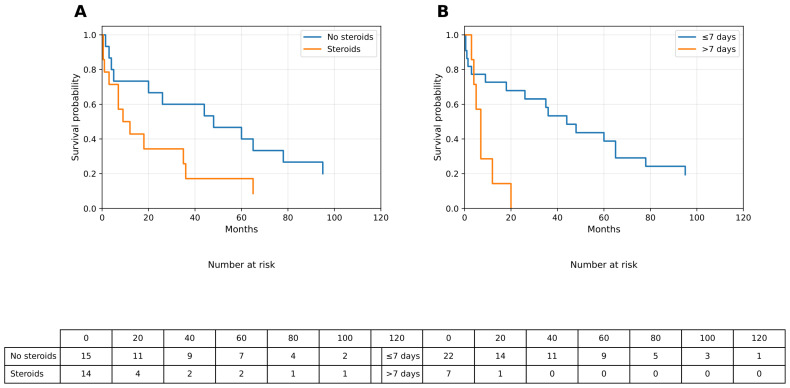
Progression-free survival according to neurosurgical workflow variables. (**A**) Kaplan–Meier curve of progression-free survival stratified by pre-biopsy corticosteroid use. (**B**) Kaplan–Meier curve of progression-free survival stratified by MRI-to-biopsy interval (≤7 days vs. >7 days). Median survival values were derived from Kaplan–Meier estimates. PFS, progression-free survival; MRI, magnetic resonance imaging.

**Progression-Free Survival and Workflow Variables:** The progression-free survival (PFS) patterns paralleled those observed for OS: median PFS was not reached in patients without pre-biopsy corticosteroid exposure, whereas patients who received steroids had a median PFS of 10.5 months, with a near-significant difference observed in log-rank testing (*p* = 0.095). Similarly, an MRI-to-biopsy interval greater than 7 days was associated with an earlier decline in PFS, whereas patients who underwent early biopsy (≤7 days) demonstrated more favorable PFS trajectories. These findings are illustrated in Figure 2A (steroid exposure) and Figure 2B (MRI-to-biopsy timing), with corresponding values provided in Table 2.

**MSKCC Prognostic Classification:** MSKCC prognostic classification demonstrated expected discriminatory performance within the cohort, supporting internal validity; median OS was not reached for Class 1 patients, whereas it was 32.5 months for Class 2 and 3 months for Class 3 patients (*p* = 0.038). A similar gradient was observed for PFS, with median values of 65, 30.5, and 1.5 months across Classes 1–3, respectively (*p* = 0.066). These results are detailed in Table 2.

**Figure 3:**
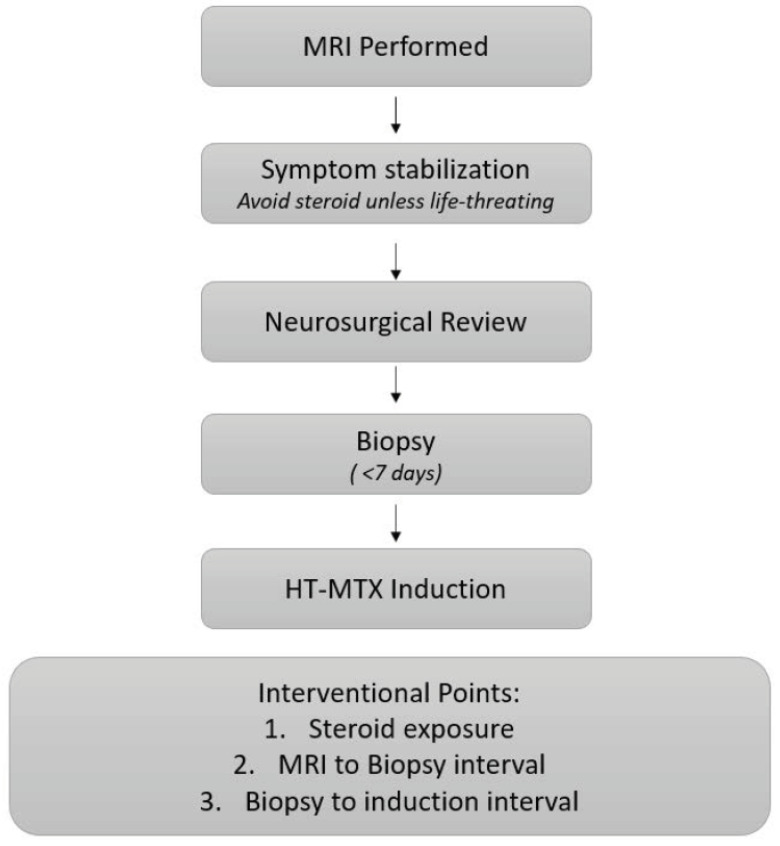
Proposed neurosurgical workflow for suspected PCNSL. Schematic representation of the recommended neurosurgical diagnostic workflow for suspected primary CNS lymphoma. Key modifiable intervention points include the following: (1) avoiding unnecessary pre-biopsy corticosteroids, (2) minimizing the MRI-to-biopsy interval, and (3) reducing the biopsy-to-induction interval.

**Workflow Variables and Survival:** A schematic overview of the neurosurgical diagnostic pathway and modifiable workflow intervals is presented in Figure 3. The flowchart illustrates the sequence from MRI acquisition to initiation of high-dose methotrexate (HD-MTX)-based therapy, highlighting two modifiable workflow intervals under neurosurgical control: pre-biopsy corticosteroid exposure and MRI-to-biopsy timing. Kaplan–Meier curves for OS and PFS based on the biopsy-to-induction interval are provided in the Supplementary Figure S1. The biopsy-to-induction interval is included for completeness, but did not demonstrate a measurable association with survival outcomes in this cohort.

## 4. Discussion

In this neurosurgical cohort, two early workflow variables that were largely under neurosurgical control demonstrated exploratory associations with survival: pre-biopsy corticosteroid exposure and MRI-to-biopsy timing. These findings suggest that potentially prognostically relevant events may also occur during the early neurosurgical phase of care, prior to oncologic treatment initiation.

Corticosteroids have historically been discussed in PCNSL primarily as diagnostic confounders, with radiological regression and pathological obscuration being well-documented results [16,17,18,19]. However, emerging translational and clinical evidence suggests that corticosteroid exposure may exert cytotoxic and cytostatic stress, alter clonal composition, and influence tumor biology even before systemic therapy is initiated [11,17,18]. These early biological pressures occur prior to initiating high-dose methotrexate (HD-MTX)-based therapy and may therefore shift the biological “starting point” at the time systemic treatment is commenced. Accordingly, empiric corticosteroid administration before diagnostic confirmation may represent an unintentional biological intervention rather than a purely symptomatic measure. At the same time, it remains plausible that the observed association between pre-biopsy corticosteroid exposure and poorer survival reflects confounding by indication, as patients requiring corticosteroids are often those presenting with greater tumor burden and mass effect or more severe neurological compromise [17,18]. Consequently, corticosteroid administration may act as a surrogate marker of an adverse baseline clinical condition rather than as an independent causal prognostic factor [11]. Consistent with this interpretation, median survival was not reached in the steroid-naïve group, whereas patients exposed to corticosteroids demonstrated markedly shorter survival trajectories. Taken together, these observations underscore the importance of accounting for initial clinical severity when evaluating early neurosurgical workflow variables in PCNSL.

The second key finding, namely that an extended MRI-to-biopsy interval was associated with less favorable survival trajectories, contributes to the mounting evidence that temporal delays are clinically relevant in PCNSL. In contrast to gliomas, which characteristically exhibit a gradual growth pattern, PCNSL is distinguished by its fast proliferation and potential for rapid radiographic changes within a short timeframe [3,6]. The authors of previous registry and real-world studies have documented that delays in the diagnosis or initiation of systemic therapy can result in adverse outcomes [12,13,19]. Our findings extend this concept by suggesting that delays occurring entirely within the neurosurgical diagnostic workflow—prior to oncologic involvement—may already be associated with early survival patterns. In this cohort, median overall survival was not reached among patients undergoing biopsy within 7 days of MRI, whereas delayed biopsy was associated with substantially shorter survival. In practical terms, the scheduling of biopsies, delays related to consultation pathways, or prolonged steroid stabilization may impose a biologically unfavorable waiting period.

In contrast, the biopsy-to-induction interval did not demonstrate a consistent association with early survival endpoints in this cohort, though this finding should be interpreted cautiously, given the small sample size and clustering of most cases within a narrow time window (median of 6 days, range of 2–15 days); this observation is more likely reflective of limited statistical power than true biological neutrality. Moreover, initiation of systemic therapy cannot occur until pathological confirmation is obtained, inherently constraining this interval in routine clinical practice. For completeness, Kaplan–Meier curves for OS and PFS according to the biopsy-to-induction interval are provided as Supplementary Material. Future prospective studies with standardized timestamp capture will be required to determine whether further acceleration of this interval confers additional survival benefits.

It is evident that neurosurgeons do not engage in the long-term oncological management of PCNSL; however, it is therefore paradoxical that neurosurgeons, rather than oncologists, often control the earliest diagnostic phase of the disease. The discrepancy between perceived and actual influence is clinically relevant, as decisions with the highest potential to modify early tumor conditions, such as expedited imaging, minimization of empiric corticosteroids, and accelerated biopsy, occur largely under neurosurgical agency. These workflows do not require additional resources but rather a conceptual shift toward viewing PCNSL as a time-sensitive malignancy. Consistent with contemporary neurosurgical series, our findings reinforce the notion that the primary role of surgery in PCNSL is diagnostic, whereas extensive cytoreductive resections rarely translate into durable oncologic benefit [20,21].

The findings of this study support the hypothesis that a more accelerated neuro-oncology diagnostic workflow may be beneficial. In centers where PCNSL is infrequently encountered, implementing standardized “PCNSL suspicion protocols” has been shown to facilitate expedited MRI access, avoidance of pre-biopsy corticosteroids except in life-threatening situations, and prioritization of same-week biopsy [18]. Optimizing this early diagnostic phase, therefore, represents a practical and underutilized opportunity to potentially improve outcomes without altering systemic treatment strategies.

Our findings in this study must be interpreted in light of several important limitations: Firstly, the retrospective design inherently limited the precision of temporal variables and introduced variability in documentation. In order to minimize over-fragmentation in a small cohort, the MRI-to-biopsy interval was analyzed using three pre-specified categories (0–7 days, 8–14 days, and ≥15 days), chosen to reflect clinically meaningful workflow thresholds described in prior PCNSL real-world series. Second, the dose and duration of corticosteroid exposure could not be reliably retrieved; therefore, steroid use was analyzed as a binary variable, potentially obscuring dose-dependent biological effects. As discussed above, corticosteroid exposure may also reflect confounding by indication. Third, heterogeneity in induction regimens, methotrexate dosing, and consolidative strategies limited adjustment for treatment-related factors.

The clinical relevance of this study lies precisely in PCNSL’s rarity. Even in high-volume tertiary centers, PCNSL constitutes a small fraction of oncologic neurosurgical practice, reflecting real-world conditions in which early diagnostic decisions are often made by clinicians with limited exposure to the disease. Accordingly, the modest sample size should be viewed not solely as a limitation but also as a realistic representation of clinical practice. In keeping with the study design and sample size, multivariable Cox regression was deliberately avoided due to insufficient events per variable, and all observed associations should therefore be interpreted as exploratory and hypothesis-generating rather than as evidence of independent prognostic effects. Larger prospective studies with standardized workflows and uniform treatment strategies are required to validate these findings.

## 5. Conclusions

Early neurosurgical workflow characteristics, including pre-biopsy corticosteroid exposure and diagnostic timing, may be associated with early survival trajectories in PCNSL. These findings underscore the neurosurgical entrance window as a potentially modifiable and cost-effective phase of care that merits prospective evaluation.

## Figures and Tables

**Table 1:** Baseline clinical and workflow characteristics of the cohort (n = 29).

Variable	Value
Age ≥60 Years, n (%)	17 (58.6)
Age <60 Years, n (%)	12 (41.4)
Age (years), median (range)	62 (38–79)
MSKCC Prognostic Class, n (%)	
Class 1	4 (13.8)
Class 2	18 (62.1)
Class 3	7 (24.1)
Pre-biopsy Corticosteroid Use, n (%)	
Yes	14 (48.3)
No	15 (51.7)
MRI-to-Biopsy Interval, n (%)	
≤7 days	22 (75.9)
>7 days	7 (24.1)
MRI-to-Biopsy Interval (days), median (range)	6 (1–21)
Biopsy-to-Induction Interval, n (%)	
≤7 days	21 (72.4)
>7 days	8 (27.6)
Biopsy-to-Induction Interval (days), median (range)	6 (2–15)
HD-MTX-based induction therapy	29 (100)

Baseline demographic, clinical, and neurosurgical workflow characteristics of patients with primary central nervous system lymphoma (PCNSL). Categorical variables are presented as a number (%). Time-related workflow variables are presented as median (range). Abbreviations: MSKCC, Memorial Sloan–Kettering Cancer Center; MRI, magnetic resonance imaging; HD-MTX, high-dose methotrexate.

**Table 2:** Survival outcomes according to neurosurgical workflow variables.

Variable	N	Median OS (Months)	Range (Months)	Log-Rank *p*	Median PFS (Months)	Range (Months)	Log-Rank *p*
Pre-biopsy Corticosteroid Use							
No steroids	15	NR	6–84		NR	6–78	
Steroids	14	12	3–36	0.127	10.5	2–36	0.095
MRI-to-Biopsy Interval							
≤7 days	22	NR	6–84		NR	6–78	
>7 days	7	6	3–7	0.074	4	3–7	0.083
Biopsy-to-Induction Interval							
≤7 days	21	48	6–84		36	6–78	
>7 days	8	7	3–36	0.806	7	2–36	0.865
MSKCC Prognostic Class							
Class 1	4	NR	48–84		65	36–78	
Class 2	18	32.5	6–69	0.038	30.5	6–48	0.066
Class 3	7	3	1–7		1.5	1–6	

Overall survival (OS) and progression-free survival (PFS) according to neurosurgical workflow variables. Median survival values were derived from Kaplan–Meier estimates and are presented together with their full ranges to reflect variability in this small cohort. NR indicates that median survival was not reached during follow-up. Survival comparisons were performed using the log-rank test. Abbreviations: OS, overall survival; PFS, progression-free survival; MRI, magnetic resonance imaging; MSKCC, Memorial Sloan–Kettering Cancer Center.

## Data Availability

The data presented in this study are available on reasonable request from the corresponding author due to ethical and privacy restrictions.

## Supplementary Materials

The following supporting information can be downloaded at https://www.mdpi.com/article/10.3390/curroncol33030139/s1. Figure S1: Survival according to biopsy-to-induction interval. (A) Kaplan–Meier curve of overall survival stratified by biopsy-to-induction interval (≤7 days vs. >7 days). (B) Kaplan–Meier curve of progression-free survival stratified by biopsy-to-induction interval (≤7 days vs. >7 days). Median survival values were derived from Kaplan–Meier estimates.
