# Supplementary figures and images for: Associations Between Early Neurosurgical Workflow and Survival in Primary Central Nervous System Lymphoma: A Single-Center Retrospective Study
Source: Curr Oncol. 2026 Feb 27;33(3):139. doi: 10.3390/curroncol33030139 (PMC13024903; doi:10.3390/curroncol33030139)

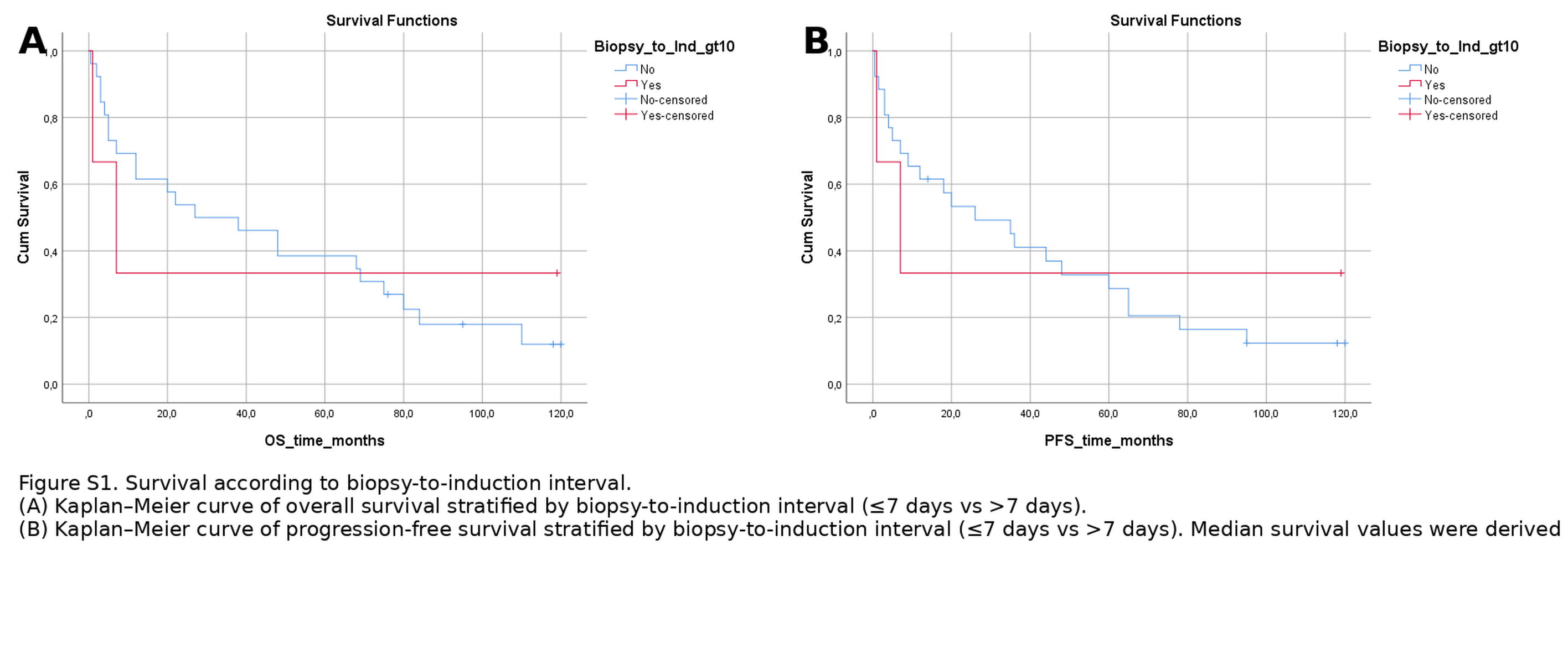

Supplement: Supplementary file 1 [file curroncol-33-00139-s001.zip › curroncol-4125128-supplementary.png]
